# Evaluation of the Potential Nephroprotective and Antimicrobial Effect of *Camellia sinensis* Leaves versus *Hibiscus sabdariffa* (*In Vivo* and *In Vitro* Studies)

**DOI:** 10.1155/2014/389834

**Published:** 2014-05-14

**Authors:** Doa'a Anwar Ibrahim, Rowida Noman Albadani

**Affiliations:** ^1^Department of Pharmacology, Faculty of Pharmacy, University of Science and Technology, Sana'a, Yemen; ^2^Department of Pharmacognosy, Faculty of Pharmacy, University of Science and Technology, Sana'a, Yemen

## Abstract

Green tea and hibiscus are widely consumed as traditional beverages in Yemen and some regional countries. They are relatively cheap and the belief is that they improve health state and cure many diseases. The aim of this study was to evaluate the potential protective and antibacterial activity of these two famous plants *in vitro* through measuring their antibacterial activity and *in vivo* through measuring nonenzymatic kidney markers dysfunction after induction of nephrotoxicity by gentamicin. Gram positive bacteria like MRSA (methicillin resistant *Staphylococcus aureus*) were isolated from hospitalized patients' different sources (pus and wound) and Gram negative bacteria including *E. coli* and *P. aeruginosa* were used *in vitro* study. In addition, the efficacy of these plants was assessed *in vivo* through measuring nonenzymatic kidney markers including S. creatinine and S. urea. Green tea was shown antimicrobial activity against MRSA with inhibition zone 19.67 ± 0.33 mm and MIC 1.25 ± 0.00 mg/mL compared with standard reference (vancomycin) 18.00 ± 0.00 mg/mL. Hibiscus did not exhibit a similar effect. Both Hibiscus- and green tea-treated groups had nephroprotective effects as they reduced the elevation in nonenzymatic kidney markers. We conclude that green tea has dual effects: antimicrobial and nephroprotective.

## 1. Introduction

In China, the usage of green tea was started for more than 4,000 years. The major species is originated from leaves of* Camellia sinensis.* Currently, this herb's use is widespread in Asia and West countries [1]. Many polyphenols are present in green tea leaves like catechins and epigallocatechin gallate. In addition, it contains tocopherols, carotenoids, ascorbic acid (vitamin C), minerals such as chromium, manganese, selenium, or zinc, and other minor phytochemical compounds [1, 2]. Consequently, animal and human studies showed that regular intake of green tea may have health benefits against cardiovascular diseases, cancer, and many types of infectious pathogens [3]. Green tea can support the immune system as well as improve the cognitive function [4].* Hibiscus sabdariffa* calyces are a famous plant cultivated and used widely in Africa, especially Egypt and Sudan as this region has a long history of the use of this drink. Hibiscus tea is known to be effective in lowering blood pressure [5] and cholesterol [6]. In addition, researchers propose that consuming hibiscus could aid in the prevention of human cancer [7].* Hibiscus sabdariffa* calyces contain different components including mucilage, polysaccharides, pectins, polyphenols, organic acids, ascorbic acid, citric, malic and tartaric acids [8].

This study aimed firstly to assess and evaluate the antibacterial activity of two plants* Camellia sinensis* leaves and* Hibiscus sabdariffa* calyces through measuring the inhibition zone of tested plants compared with antibiotic references* in vitro* study. Secondly, the study aimed to compare the potential nephroprotective effect of two tested plants in* in vivo* study.

## 2. Materials and Methods

### 2.1. Materials

#### 2.1.1. Drugs and Natural Products

Gentamicin sulfate was purchased from Alpha Aleppo pharmaceutical Ind.

Fresh green tea leaves and* Hibiscus sabdariffa* calyces were bought from special herbal stores in Sana'a city. The two plants were identified and authenticated at Pharmacognosy department, University of Science and Technology (UST).

#### 2.1.2. Animals and Bacteria

- Different bacteria species Gram positive including methicillin resistant* Staphylococcus aureus *(MRSA) and Gram negative bacteria including* Escherichia coli* and* Pseudomonas aeruginosa *were supplied by the UST hospital laboratory where these were isolated freshly from infected patients.
- Experimental animals being adult male New Zealand White Rabbits with weight range from 1–1.5 kg and age 7 months ±1 week were supplied by the Faculty animal house. They were fed with fresh green grass and housed in stainless steel cages (2500 cm^2^ with a height of 35 cm) away from direct sunlight.

### 2.2. Preparation of Extracts

The dried plants subsequently were ground separately using a blender to fine powder.

Two kilograms (2 kg) of each dried plant material (green tea and hibiscus) were introduced separately into a Whatman paper thimble and then extracted by refluxing with water as solvent system in a Soxhlet apparatus for six hours. A second extraction was made using 80% methanol and concentrated using a rotary evaporator at 40°C and finally dried in a vacuum dissector at 40°C. The resulting residue which weighed 32.52 g (recovery 10.33%) was later stored under 4°C until required. A 10 mg/mL solution of the extract was prepared in distilled water before administration to the rabbits [9].

### 2.3. Determination of Antibacterial Activity

Disc diffusion assay [10] was used to determine the antimicrobial activity of the investigated extracts. Sterile filter paper discs of 6 mm diameter were impregnated with 35 *μ*L of the extracted solution (equivalent to 7 mg of dried extract). The paper's discs were allowed to evaporate and then placed on the surface of agar plates that were previously inoculated with microbial cell. Plates were kept for 2 hours in refrigerator to enable prediffusion of extract into the agar. The plates then were incubated overnight (18 hours) at 37°C. Vancomycin, co-trimoxazole, and piperacillin were used as positive control for methicillin resistant* Staphylococcus aureus*,* Escherichia coli,* and* Pseudomonas aeruginosa,* respectively. At the end of incubation period, the antibacterial activity was evaluated by measuring the inhibition zones (diameter of inhibition zone plus diameter of the disc). An inhibition zone of 14 mm or greater is considered as high antibacterial activity [10].

### 2.4. Minimum Inhibitory Concentration

According the method of Olaleye and Mary Tolulope, 2007, serial dilution of plant extract was used: 20, 10, 5, 2.5, 1.25, 0.625, and 0.313 mg/mL. Each mL (innocula) was poured into petri dish and allowed to set after the agar was also poured. A 3 mm sterile cork borer was used to make wells. The serial freshly prepared dilutions were poured into these wells. The plates were incubated at 37°C for 24 h. Finally, the growth of tested microorganisms (m.o) was observed and compared with clear zone. The least concentration of plant extract that inhibits growth of tested m.o is considered as minimum inhibitory concentration (MIC) [11].

### 2.5. Animal Study Design

Twenty-four rabbits were divided into four groups randomly. Each group contained six animals. Before starting the experiment, all animals were kept for five days to be acclimatized.

Nephrotoxicity was induced in groups II, III, and IV intraperitoneally by gentamicin (80 mg/kg body weight) for seven consecutive days [12, 13]. Dose of 250 mg/kg body weight, of the* Hibiscus sabdariffa *calyx extract was administered to rabbits in group III, and 300 mg/kg body weight, of* Camellia sinensis* was given to rabbits in group IV, respectively. Rabbits in group I were given distilled water and kept as a control. All groups received tested agents via an oral route using a gavage needle once daily for seven days.

At the end of experiment, light anesthesia with halothane was used, and the animals were dissected. Blood samples were collected directly through the cardiac puncture and kept in container free from anticoagulant and allowed to clot for 20 minutes and centrifuged at 4000 rpm for 15 minutes.

Sera were collected using micropipettes and analyzed. Nonenzymatic markers of kidney dysfunction were measured including serum creatinine [14] and urea [15]. All the experimental procedures were in accordance with the guidelines for the care and use of laboratory animals, and approval from the Institutional Research and Ethics Committee, UST, was received prior to the experiments.

### 2.6. Statistical Analysis

Results of this work were expressed as mean ± standard error of the mean (S.E.M) by using the Statistical Analysis (SPSS) software package version 18.0. ANVOA was used to compare between groups. *P* < 0.05 was considered as significant.

## 3. Results

In* in vitro* study, water and methanolic extracts of both* Camellia sinensis* and* Hibiscus sabdariffa* were tested against MRSA (pus), MRSA (wound),* E. coli*, and* P. aeruginosa* and compared with the documented references. Only water and methanolic extracts of* Camellia sinensis* were shown 21, 22, 18, and 19 mm inhibiting zones, respectively. Extracts of* Hibiscus sabdariffa* did not produce antibacterial activity against tested bacterial species. This experiment was repeated three times to get the average inhibition zone of the tested extracts as shown in (Tables 1 and 2).

**Figure 1:**
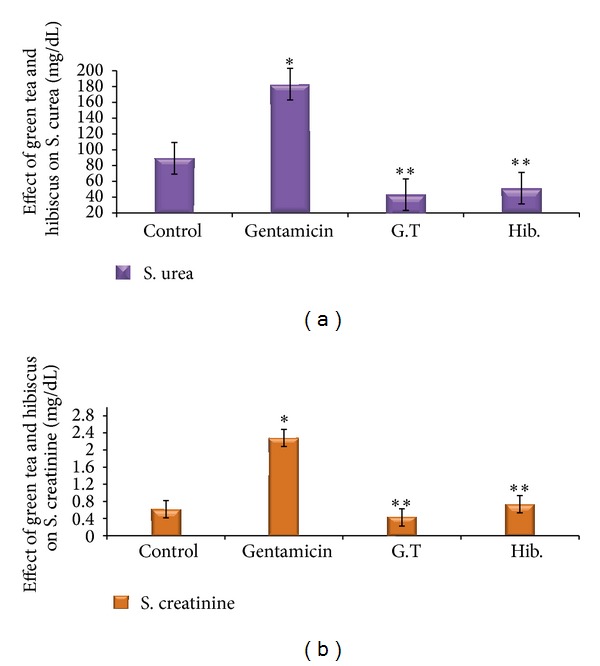
(a) Effect of green tea (300 mg/d) and hibiscus (250 mg/d) on the (mean ± SE) nonenzymatic markers of kidney dysfunction (serum urea mg/dL) for 7 days in adult rabbits (*n* = 6). *Significant as compared with control at *P* value < 0.05. **Significant as compared with gentamicin-induced nephrotoxicity at *P* value < 0.05. G.T: green tea, Hib: hibiscus. (b) Effect of green tea (300 mg/d) and hibiscus (250 mg/d) on the (mean ± SE) nonenzymatic markers of kidney dysfunction (serum creatinine mg/dL) for 7 days in adult rabbits (*n* = 6). *Significant as compared with control at *P* value < 0.05. **Significant as compared with gentamicin-induced nephrotoxicity at *P* value < 0.05. G.T: green tea, Hib: hibiscus.

Regarding the* in vivo* study, levels of creatinine and urea in sera, gentamicin group displayed significant increase in urea (110.5 ± 17.52 mg/dL) and creatinine (1.62 ± 0.72 mg/dL) compared with control group. However, the green tea-treated group demonstrated significant reduction in urea (43.83 ± 3.45 mg/dL) and creatinine (0.617 ± 0.167 mg/dL) compared with gentamicin group. The hibiscus-treated group also revealed significant reduction in both nonenzymatic markers of kidney dysfunctions urea (43.30 ± 6.47 mg/dL) and creatinine (0.733 ± 0.114 mg/dL) compared with gentamicin group. There was insignificant difference between two tested plants as shown in (Figures 1(a) and 1(b)).

## 4. Discussion

*Staphylococcus aureus* is a contagious type of bacteria that can cause serious infection, especially nosocomial infections [16]. In the present study, both methanolic and water extracts of plant* Camellia sinensis* have been tested. The extracts displayed antimicrobial effect especially against Gram-positive bacteria as they inhibited the growth of methicillin resistant* Staph. aureus*. The antimicrobial effect of green tea extract is similar to that of the reference used (vancomycin), although it had no activity against gram negative bacteria like* E. coli* and* P. aeruginosa*. Hamilton-Miller, 1995, and Yamada et al., 2006, supported our findings. They found that green tea catechins possess strong antibacterial activity [17, 18].

The antimicrobial activity of green tea (*Camellia sinensis*) against MRSA may be related to the presence of epicatechin gallate (ECG) and epigallocatechin gallate (EGCG). These two polyphenols that are abundant in green tea (*Camellia sinensis*) were identified as candidates for treating* S. aureus*. Chromosomal factors, known as “fem factors”, are links between branched-chain cell wall peptide formation and *β*-lactam rings that are necessary for* S. aureus*, to show resistance to methicillin. Scientists have identified ECG as a potential inhibitor of these fem factors. In addition, ECG also has been recognized as a selective growth inhibitor of MRSA because it damages the cell wall of the bacteria. It is only effective when ECG is present in high concentrations [19–22].

However, this study showed that methanolic and water extracts of hibiscus flowers were free from antimicrobial activity being neither against Gram-positive nor against Gram-negative bacteria. Our result disagreed with Mahadevan et al., 2009 and VimalinHena, 2010, who found that the organism* Staphylococcus aureus* is sensitive towards both the leaves and flowers hot aqueous extract of hibiscus. This effect is due to the polyphenolic nature of the flavonoid gossypetin [23, 24].

Antioxidant compounds are very important for inhibition of oxidative damage to biological target molecules. They are used for prevention and treatment of many diseases such as cancer and cardiovascular, autoimmune, and neurodegenerative diseases as well as inflammatory effect [25]. Gentamicin was used in this study to induce kidney dysfunction as a model of nephrotoxicity. Silan et al. 2007 and Soliman et al. 2007 supported our findings. They showed that gentamicin produced an elevation in the concentrations of biochemical indicators of kidney function such as blood urea nitrogen (BUN) and serum creatinine [26]. Both green tea- and hibiscus-treated group had shown significant nephroprotective effects. They reduced biochemical indicators or nonenzymatic markers of the kidney dysfunction compared with gentamicin-induced nephrotoxicity. Okoko and Oruambo 2008 agree with our outcomes. They found that* Hibiscus sabdariffa* extract reduced the levels of serum creatinine, urea, and the elevation of the levels of kidney GSH and catalase in rats [27].

The exact mechanism of action is not clear. There are many suggestions; the extract of this plant may lower the level of lipid peroxidation that is elevated in response to some toxic materials like gentamicin. Another suggestion points toward the interaction between the phytochemicals and the toxic materials. In addition, calyces of* Hibiscus sabdariffa* contain potent antioxidant components including vitamin C and tocopherol. This explains the protective effect of this plant as it functions in the conversion of *α*-tocopheroxy radical to *α*-tocopherol or reduction of Ca^+2^ dependent permeabilization of renal cortex mitochondria [27–29]. On the other hand, antioxidant and radical scavenger of these calyces are related to the presence of flavonoids known as anthocyanins [27, 30, 31].

Koyner et al., 2008 and Ali et al., 2011, showed that several extracts of medicinal plants including green tea have been tested against gentamicin-induced nephrotoxicity in rats. The basis of the protective action of those plant extracts is not known with certainty, but it was thought to be directly related to their antioxidant properties [32, 33]. In addition, Kadkhodaee et al., 2005 and Kadkhodaee et al., 2007, found that the nephroprotective effect of green tea may be due to the presence of vitamins C and E in its composition [34, 35]. As previously noted, green catechins can act as scavengers of free radicals caused by reactive oxygen species and prevent free radical damage [36].

## 5. Conclusion

From this study, we conclude that green tea is a good and potent antimicrobial agent, mainly against MRSA, since it has a superior effect than hibiscus in this manner. According to their potential nephroprotective effect, both plants showed very close efficacy without any significant difference between them in their ability to ameliorate nephrotoxicity induced by gentamicin. This may due to the potent antioxidant activity of these two plants. Further studies are needed to elucidate the presence of other components in green tea and hibiscus that may have other health benefits such as anticancer or neuroprotective effects.

## Figures and Tables

**Table 1:** Antibacterial activity of water and methanolic extracts of *Camellia sinensis* and *Hibiscus sabdariffa *on tested Gram positive and Gram negative bacteria.

Bacteria species	Reference	T.w	T.m	H.w	H.m
MRSA (pus)	Van. [19]	21	22	R	10
MRSA (wound)	Van. [17]	18	19	R	R
*E. coli *	GT. [33]	R	R	R	R
*P. aeruginosa *	Pi. [30]	R	R	R	11

T.w: water extract of green tea; T.m: methanolic extract of green tea; H.w: water extract of hibiscus; H.m: methanolic extract of hibiscus; Van.: vancomycin; GT: gentamicin; Pi: piperacillin; R: resistance.

**Table 2:** Detection of MIC (mg/mL) of methanolic extract of *Camellia sinensis* by using MRSA (wound) species (*n* = 3).

Bacteria	Inhibition zone (mm)		MIC (mg/mL)
	10 mg/L	Van. 1 mg/L	G.T
*Staph. aureus *	19.67 ± 0.33	18.00 ± 0.00	1.25 ± 0.00
*E. coli *	0	0	0
*P. aeruginosa *	0	0	0

Results are expressed as mean ± SE; *n* = 3; MIC: minimum inhibitory concentration; G.T: green tea; Van.: vancomycin.
